# Comparison of thromboelastographic profiles and clinical characteristics in children with severe Mycoplasma pneumoniae pneumonia

**DOI:** 10.3389/fped.2025.1459455

**Published:** 2025-01-22

**Authors:** Yong-tao Li, Yu-mei Ma, Fu-li Dai, Zhen Peng, Ya-ping Dai, Ke Zhi, Hai-hong Feng, Shu-jun Li

**Affiliations:** ^1^ Department of Pediatrics, Luoyang Maternal and Child Health Hospital, Luoyang, Henan, China; ^2^ Department of Pediatrics, The First Affiliated Hospital of Xinxiang Medical University, Xinxiang, Henan, China

**Keywords:** thromboelastographic profiles, clinical characteristics, mycoplasma pneumoniae pneumonia, severe, children

## Abstract

**Background:**

This study aimed to compare Thromboelastographic (TEG) profiles and clinical characteristics between severe Mycoplasma pneumoniae (MP) pneumonia patients with normal and abnormal TEG parameters.

**Methods:**

The clinical data of 133 children with severe MP pneumonia were retrospectively analyzed. Patients were divided into normal (*n* = 76) and abnormal (*n* = 57) TEG groups. Demographic characteristics, clinical manifestations, laboratory findings, imaging features, bronchoscopy results, treatment, complications, and outcomes were compared between groups.

**Results:**

The abnormal TEG group (42.9%) had longer fever duration (median: 8.5 vs. 7.0 days, *P* < 0.001) and hospital stay (median: 11.5 vs. 10.0 days, *P* = 0.003). They also showed higher levels of C-reactive protein (median: 30.2 vs. 20.1 mg/L, *P* < 0.001), lactate dehydrogenase (median: 334.5 vs. 276.0 U/L, *P* = 0.001), and D-dimer (median: 1.2 vs. 0.5 μg/ml, *P* < 0.001). HRCT revealed more lobar consolidation or multilobar involvement (36.8% vs. 18.4%, *P* = 0.016), and bronchoscopy showed more mucous plug obstruction (28.1% vs. 10.5%, *P* = 0.008) in the abnormal TEG group. TEG parameters indicated a hypercoagulable state with shorter R time (*P* < 0.001), shorter K time (*P* < 0.001), and higher MA (*P* = 0.003). The abnormal TEG group had higher incidences of coagulopathy (*P* < 0.001), cardiac involvement (elevated cardiac enzymes: 36.8% vs. 17.1%, *P* = 0.009; pericardial effusion: 10.5% vs. 1.3%, *P* = 0.017), and plastic bronchitis (*P* = 0.006). They also required longer azithromycin courses (median: 15 vs. 14 days, *P* = 0.026).

**Conclusion:**

Children with severe MP pneumonia and abnormal TEG profiles have more severe clinical manifestations, higher inflammatory markers, more extensive lung involvement, and a higher incidence of complications. TEG may help identify high-risk patients and guide management in severe MP pneumonia.

## Introduction

Mycoplasma pneumoniae (MP) is a common cause of community-acquired pneumonia in children, accounting for 10%–40% of cases (1). Although most MP pneumonia cases are self-limited, severe cases can lead to various complications, such as necrotizing pneumonia, lung abscess, pleural effusion, and even acute respiratory distress syndrome (ARDS) (2). Coagulopathy is another potential complication of severe MP pneumonia, which may contribute to the severity of the disease and poor outcomes (3).

Thromboelastography (TEG) is a point-of-care test that provides a comprehensive assessment of the global coagulation function, including clot formation, strength, and stability (4). TEG is particularly valuable in diagnosing and managing coagulation disorders because it offers real-time insights into the clotting process, which can be crucial for identifying hypercoagulable states and guiding treatment strategies. Previous studies have demonstrated the utility of TEG in identifying hypercoagulable states in various diseases, such as sepsis, trauma, and malignancy (5–7). Despite these applications, the role of TEG in evaluating coagulation function in children with severe MP pneumonia remains unclear. Understanding how TEG profiles correlate with disease severity and outcomes could provide important insights into the management of severe MP pneumonia and improve patient care.

In the context of MP diagnosis, polymerase chain reaction (PCR) testing is the standard method for confirming the presence of MP, given its high sensitivity and specificity. However, while PCR is essential for diagnosing MP, TEG could offer additional diagnostic value by revealing coagulation abnormalities that are not apparent through PCR alone. TEG's role is to complement PCR by providing insights into the patient's coagulation status, which is critical for managing severe cases.

Additionally, the availability and cost of TEG are important considerations. TEG is a specialized test that might not be available in all healthcare settings, and its cost can vary significantly. Addressing these factors is crucial for assessing the feasibility of implementing TEG as part of routine care for severe MP pneumonia.

This study aimed to compare the TEG profiles and clinical characteristics between children with severe MP pneumonia who had normal and abnormal TEG parameters. We hypothesized that patients with abnormal TEG profiles would have more severe clinical manifestations, higher levels of inflammatory markers, more extensive lung involvement, and a higher incidence of complications compared to those with normal TEG profiles.

## Methods

### Study design and participants

We conducted a retrospective study of children with severe MP pneumonia admitted to our hospital between December 2023 and March 2024. The patient's diagnosis is made by the attending physician and confirmed by the three-level physician ward round system. The diagnosis of severe Mycoplasma pneumonia is based on the Guidelines for the Diagnosis and Treatment of Mycoplasma Pneumonia in Children (2023 Edition) (8) and the Standards for the Diagnosis and Treatment of Community-Acquired Pneumonia in Children (2019 Edition) (9). The inclusion criteria were: (1) age ≤18 years; (2) diagnosis of severe MP pneumonia based on clinical manifestations, radiological findings, and positive MP polymerase chain reaction (PCR) tests from nasopharyngeal swabs or bronchoalveolar lavage fluid (BALF); and (3) TEG performed within 24 h of admission. The exclusion criteria were: (1) co-infection with other pathogens; (2) underlying chronic diseases; and (3) incomplete clinical data.

The study protocol was approved by the Ethics Committee of our hospital, and informed consent was obtained from the parents of all participants. Bronchoscopy was performed after obtaining written informed consent from the parents.

### Data collection

Demographic characteristics, clinical manifestations, laboratory findings, imaging features, bronchoscopy results, treatment, complications, and outcomes were collected from electronic medical records.

The TEG test was performed within 24 h of admission using the TEG 5000 analyzer with standard detection. The procedure involved drawing 1 ml of whole blood from a citrate anticoagulation tube into a Kaolin tube, mixing it, and letting it stand for 2–3 min. Then, 340 µl of activated whole blood was transferred to a white sample cup pre-filled with 20 µl of 0.2 mol/L calcium chloride solution. The sample type selected was CK. After testing, the TEG parameters, including reaction time (R), clot formation time (K), α-angle, maximum amplitude (MA), and lysis at 30 min (LY30), were automatically recorded.

### Definitions

Severe MP pneumonia was defined as meeting any of the following criteria: (1) persistent high fever (≥39°C) for ≥5 days or fever for ≥7 days with no improvement in peak body temperature; (2) rapid progression of lung lesions (>50% increase within 48 h) on chest imaging; (3) dyspnea, respiratory distress, or hypoxemia requiring oxygen therapy; or (4) presence of complications such as pleural effusion, lung abscess, necrotizing pneumonia, or ARDS (10).

Abnormal TEG was defined as having any of the following parameters outside the normal reference range: (1) shortened R time: <4.5 min, indicating a hypercoagulable state; (2) prolonged R time: >8 min, indicating a hypocoagulable state or the use of anticoagulants; (3) shortened K time: <1 min; (4) prolonged K time: >3 min; (5) increased α-angle: >60 degrees; (6) decreased α-angle: <45 degrees; (7) increased MA: >70 mm; or (8) decreased MA: <50 mm (11); (9) LY30 (Lysis at 30 min): usually less than 8%, indicating the degree of dissolution within 30 min after thrombus formation.

Coagulopathy was defined as having any of the following criteria: (1) thrombin time (TT) prolonged by >3 s; (2) prothrombin time (PT) prolonged by >3 s; (3) activated partial thromboplastin time (APTT) prolonged by >10 s; or (4) fibrinogen (FIB), fibrin degradation products (FDP), or D-dimer outside the normal range (12).

### Statistical analysis

Continuous variables were expressed as median (interquartile range) and compared using the Mann–Whitney *U* test. This test was used for continuous variables due to their non-normal distribution. Unlike parametric tests that assume normality, the Mann–Whitney *U* test is a non-parametric test that compares the ranks of values between two independent groups, making it suitable for non-normally distributed data. These tests were used for categorical variables to compare proportions between groups. However, when dealing with smaller sample sizes or when the expected frequency in any cell was less than 5, Fisher's exact test was applied to ensure accurate results. Categorical variables were expressed as number (percentage) and compared using the chi-square test or Fisher's exact test. A two-tailed *P*-value < 0.05 was considered statistically significant. All statistical analyses were performed using SPSS version 25.0 (IBM Corp., Armonk, NY, USA).

## Results

### Demographic and clinical characteristics

A total of 133 children with severe MP pneumonia were included in the analysis, with 76 (57.1%) in the normal TEG group and 57 (42.9%) in the abnormal TEG group. The median age was 7.1 years (range: 4.3–9.3 years) in the normal TEG group and 6.7 years (range: 5.2–8.0 years) in the abnormal TEG group (*P* = 0.135). There were no significant differences in sex, underlying diseases, or duration of symptoms before admission between the two groups (Table 1).

**Table 1:** Demographic and clinical characteristics of children with severe Mycoplasma pneumoniae pneumonia.

Characteristic	Normal TEG (*n* = 76)	Abnormal TEG (*n* = 57)	Z value	*P*-value
Age (years)	7.1 (4.3–9.3)	6.7 (5.2–8.0)	−1.49	0.135
Male (%)	41 (53.9%)	33 (57.9%)	–	0.646
Underlying diseases (%)	12 (15.8%)	6 (10.5%)	–	0.379
Duration of symptoms before admission (days)	5 (3–7)	6 (4–8)	−1.90	0.057
Duration of fever (days)	7.0 (5.0–9.0)	8.5 (7.0–12.0)	−4.23	<0.001
Length of hospital stay (days)	10.0 (8.0–13.0)	11.5 (9.0–15.0)	−2.98	0.003

Data are presented as median (interquartile range) or number (percentage).

The abnormal TEG group had a significantly longer duration of fever (median: 8.5 vs. 7.0 days, *P* < 0.001) and hospital stay (median: 11.5 vs. 10.0 days, *P* = 0.003) compared to the normal TEG group (Table 1).

### Laboratory findings

The abnormal TEG group had significantly higher levels of C-reactive protein (CRP) (median: 30.2 vs. 20.1 mg/L, *P* < 0.001), lactate dehydrogenase (LDH) (median: 334.5 vs. 276.0 U/L, *P* = 0.001), and D-dimer (median: 1.2 vs. 0.5 μg/ml, *P* < 0.001) compared to the normal TEG group. There were no significant differences in white blood cell count, neutrophil percentage, or procalcitonin levels between the two groups (Table 2).

**Table 2:** Laboratory findings of children with severe Mycoplasma pneumoniae pneumonia.

Variable	Normal TEG (*n* = 76)	Abnormal TEG (*n* = 57)	*P*-value
White blood cell count (×10^9 ^/L)	7.8 (5.9–10.4)	8.2 (5.7–11.5)	0.461
Neutrophil percentage (%)	65.0 (56.0–74.0)	68.0 (59.0–77.0)	0.231
C-reactive protein (mg/L)	20.1 (8.7–31.5)	30.2 (17.6–42.8)	<0.001
Procalcitonin (ng/ml)	0.2 (0.1–0.5)	0.3 (0.1–0.8)	0.114
Lactate dehydrogenase (U/L)	276.0 (234.0–318.0)	334.5 (288.0–381.0)	0.001
D-dimer (μg/ml)	0.5 (0.3–0.8)	1.2 (0.6–1.8)	<0.001

Data are presented as median (interquartile range).

### Imaging features and bronchoscopy findings

High-resolution computed tomography (HRCT) showed lobar consolidation (≥2/3 of a lobe) or multilobar involvement more frequently in the abnormal TEG group (36.8% vs. 18.4%, *P* = 0.016).

Bronchoscopy was performed in 79 (59.4%) patients, with 32 (42.1%) in the normal TEG group and 47 (82.5%) in the abnormal TEG group. Mucous plug obstruction requiring removal was more frequently observed in the abnormal TEG group (28.1% vs. 10.5%, *P* = 0.008) (Table 3).

**Table 3:** Imaging features and bronchoscopy findings of children with severe Mycoplasma pneumoniae pneumonia.

Variable	Normal TEG (*n* = 76)	Abnormal TEG (*n* = 57)	*P*-value
Lobar consolidation or multilobar involvement	14 (18.4%)	21 (36.8%)	0.016
Mucous plug obstruction	8 (10.5%)	16 (28.1%)	0.008

Data are presented as number (percentage).

### TEG parameters and coagulation function

The abnormal TEG group had significantly shorter R time (median: 5.6 vs. 6.6 min, *P* < 0.001), shorter K time (median: 1.7 vs. 2.0 min, *P* < 0.001), and higher MA (median: 63.3 vs. 60.8 mm, *P* = 0.003) compared to the normal TEG group. There were no significant differences in α-angle or LY30 between the two groups (Table 4). The incidence of coagulopathy was significantly higher in the abnormal TEG group (40.4% vs. 10.5%, *P* < 0.001). However, there were no differences in the levels of PT, APTT, TT, FIB and FDP between different patients (all *P* > 0.05) (Table 4).

**Table 4:** TEG parameters and coagulation function of children with severe Mycoplasma pneumoniae pneumonia.

Variable	Normal TEG (*n* = 76)	Abnormal TEG (*n* = 57)	*P*-value
R time (min)	6.6 (5.9–7.3)	5.6 (4.8–6.4)	<0.001
K time (min)	2.0 (1.8–2.3)	1.7 (1.4–2.0)	<0.001
α-angle (degrees)	60.2 (58.1–64.3)	60.4 (57.3–63.5)	0.473
MA (mm)	60.8 (56.5–65.1)	63.3 (59.5–68.3)	0.003
LY30 (%)	1.5 (0.4–2.6)	1.6 (0.7–2.5)	0.772
Coagulopathy	8 (10.5%)	23 (40.4%)	<0.001
PT (s)	13.1 (12.5–13.6)	13.2 (12.7–13.8)	0.814
APTT (s)	28.3 (25.5–32.5)	29.3 (25.7–33.6)	0.108
TT (s)	16.3 (15.8–16.9)	16.1 (15.7–16.7)	0.579
FIB (g/L)	4.34 (3.91–4.74)	4.39 (4.00–4.62)	0.884
FDP (μg/ml)	1.37 (1.05–2.21)	1.27 (1.02–2.21)	0.097

Data are presented as median (interquartile range) or number (percentage).

### Treatment and outcomes

All patients received azithromycin as the initial antibiotic treatment. The abnormal TEG group required a longer duration of azithromycin treatment compared to the normal TEG group (median 15 days, range: 14–21 days vs. median 14 days, range: 10–21 days; *P* = 0.026). Corticosteroids were used in 58 (43.6%) patients, with no significant difference between the normal TEG group (40.8%) and the abnormal TEG group (47.4%, *P* = 0.447) (Table 5).

**Table 5:** Treatment and outcomes of children with severe Mycoplasma pneumoniae pneumonia.

Variable	Normal TEG (*n* = 76)	Abnormal TEG (*n* = 57)	*P*-value
Duration of azithromycin treatment (days)	14 (10–21)	15 (14–21)	0.026
Corticosteroid use	31 (40.8%)	27 (47.4%)	0.447
Plastic bronchitis	5 (6.6%)	13 (22.8%)	0.006
Necrotizing pneumonia	3 (3.9%)	5 (8.8%)	0.235
Lung abscess	2 (2.6%)	4 (7.0%)	0.212
Elevated cardiac enzymes	13 (17.1%)	21 (36.8%)	0.009
Pericardial effusion	1 (1.3%)	6 (10.5%)	0.017
Obliterative bronchiolitis	2 (2.6%)	5 (8.8%)	0.118
Atelectasis	3 (3.9%)	7 (12.3%)	0.065
Bronchiectasis	1 (1.3%)	3 (5.3%)	0.173

Data are presented as median (interquartile range) or number (percentage).

Complications were more frequent in the abnormal TEG group. Plastic bronchitis was diagnosed significantly more often in the abnormal TEG group compared to the normal TEG group (22.8% vs. 6.6%, *P* = 0.006). The abnormal TEG group also showed a higher incidence of cardiac involvement (Cardiac involvement mainly includes: significantly elevated myocardial enzyme spectrum, pericardial effusion, arrhythmia, etc.), as evidenced by elevated cardiac enzymes (36.8% vs. 17.1%, *P* = 0.009) and pericardial effusion (10.5% vs. 1.3%, *P* = 0.017) (Table 5).

Long-term pulmonary complications were observed in both groups, although the differences did not reach statistical significance. Obliterative bronchiolitis was more common in the abnormal TEG group (8.8% vs. 2.6%, *P* = 0.118), as was atelectasis (12.3% vs. 3.9%, *P* = 0.065) and bronchiectasis (5.3% vs. 1.3%, *P* = 0.173) (Table 5).

## Discussion

Severe mycoplasma pneumonia (MP) is associated with coagulation dysfunction and thromboembolic diseases, such as pulmonary embolism and cerebral infarction, which can lead to poor outcomes. This study demonstrated that children with severe MP and abnormal TEG profiles exhibited more severe clinical manifestations, higher levels of inflammatory markers, greater lung involvement, and a higher incidence of complications compared to those with normal TEG profiles. To the best of our knowledge, this is the first study to directly compare TEG profiles and clinical characteristics in children with severe MP pneumonia.

Our findings showed that children with severe MP pneumonia and abnormal TEG profiles exhibited a hypercoagulable state, as evidenced by shorter R and K times and higher MA values compared to those with normal TEG profiles. This observation is consistent with previous studies, such as that of Zhang et al. (13), who reported significant coagulation abnormalities in children with severe MP pneumonia, characterized by elevated D-dimer levels and prolonged conventional coagulation times; however, their study did not employ TEG to evaluate global coagulation function. The hypercoagulable state in the abnormal TEG group may be attributed to the severe inflammatory response in these patients (14, 15), as reflected by higher levels of CRP, LDH, and D-dimer, which are known to activate the coagulation cascade and promote hypercoagulability (16, 17). This condition may contribute to the development of complications such as mucous plug obstruction and plastic bronchitis. By incorporating TEG, our study provided a more comprehensive evaluation of coagulation function, offering new insights into the mechanisms underlying coagulation abnormalities in severe MP pneumonia.

It is noteworthy that the inflammatory response and immune dysregulation induced by MP infection may indirectly affect TEG parameters. Inflammation can increase platelet activation, influencing measurements such as MA (maximum amplitude) and Ly30 (lysis at 30 min). Elevated inflammatory markers are often associated with heightened platelet activation, which may enhance clot strength (reflected by MA) and alter clot stability (affecting Ly30), partially explaining the hypercoagulable state observed in patients with abnormal TEG profiles. In our clinical practice, we found that some children with severe MP pneumonia exhibited TEG abnormalities within the early stages of the disease (especially within the first five days), indicating a hypercoagulable state. These patients often presented with persistent high fever and poor general condition, even when other inflammatory markers (e.g., neutrophil percentage, CRP, LDH) and chest imaging did not indicate severe infection. However, bronchoscopy frequently revealed mucous plug obstruction. Early treatment of these patients as severe infections resulted in favorable outcomes and reduced the risk of sequelae. These findings underscore the potential value of TEG in the early identification of high-risk patients and in guiding timely and appropriate treatment.

Our study also revealed an association between abnormal TEG profiles and complications such as plastic bronchitis and cardiac involvement. Notably, the higher incidence of plastic bronchitis in the abnormal TEG group is particularly concerning. Plastic bronchitis, a rare but potentially life-threatening condition, is characterized by the formation of cohesive, branching airway casts (18). The hypercoagulable state observed in patients with abnormal TEG profiles may facilitate the formation of these bronchial casts by promoting fibrin deposition in the airways. This finding highlights the potential benefit of early anticoagulation therapy in preventing plastic bronchitis among high-risk patients. Equally concerning is the association between abnormal TEG profiles and cardiac involvement. Previous studies have shown that MP infection can lead to various cardiac complications, including myocarditis, pericarditis, and arrhythmias (19). The hypercoagulable state, coupled with an intensified inflammatory response in patients with abnormal TEG profiles, may contribute to these cardiac complications. These observations underscore the importance of vigilant cardiac monitoring in patients with severe MP pneumonia, particularly those exhibiting abnormal TEG parameters, to enable timely intervention and reduce the risk of severe outcomes.

This study has several limitations. First, its retrospective design may introduce selection and information biases. Second, the single-center setting may limit the generalizability of the findings to broader populations. Third, the absence of follow-up TEG data prevents an evaluation of dynamic changes in coagulation function over the course of the disease. Fourth, the lack of a control group comprising patients with non-severe MP pneumonia restricts our ability to determine whether TEG abnormalities are specific to severe cases or also occur in milder infections. Future research should focus on multicenter, prospective studies with longitudinal TEG assessments and include a broader spectrum of disease severity to better clarify the clinical significance of TEG abnormalities in MP pneumonia.

## Conclusion

In conclusion, children with severe MP pneumonia and abnormal TEG profiles have more severe clinical manifestations, higher inflammatory markers, more extensive lung involvement, and a higher incidence of complications compared to those with normal TEG. TEG may be a useful tool for identifying high-risk patients and guiding management in severe MP pneumonia. Further studies are needed to investigate the role of TEG in predicting outcomes and informing treatment decisions in this population.

## Data Availability

The original contributions presented in the study are included in the article/Supplementary Material, further inquiries can be directed to the corresponding author.

## References

[1] Poddighe D. Mycoplasma pneumoniae-related hepatitis in children. Microb Pathog. (2020) 139:103863. 10.1016/j.micpath.2019.103863 31712120

[2] Lee KL Lee CM Yang TL Yen TY Chang LY Chen JM Severe Mycoplasma pneumoniae pneumonia requiring intensive care in children, 2010–2019. J Formos Med Assoc. (2021) 120(1 Pt 1):281–91. 10.1016/j.jfma.2020.08.018 32948415

[3] Zheng Y Hua L Zhao Q Li M Huang M Zhou Y The level of D-dimer is positively correlated with the severity of *Mycoplasma pneumoniae* pneumonia in children. Front Cell Infect Microbiol. (2021) 11:687391. 10.3389/fcimb.2021.687391 34336714 PMC8319762

[4] Wagner ML Johnston M Jenkins T Palumbo JS Rymeski BA. Use of thromboelastography in children on extracorporeal membrane oxygenation. J Pediatr Surg. (2022) 57(6):1056–61. 10.1016/j.jpedsurg.2022.01.059 35304022

[5] Haase N Ostrowski SR Wetterslev J Lange T Møller MH Tousi H Thromboelastography in patients with severe sepsis: a prospective cohort study. Intensive Care Med. (2015) 41(1):77–85. 10.1007/s00134-014-3552-9 25413378

[6] Baksaas-Aasen K Gall LS Stensballe J Juffermans NP Curry N Maegele M Viscoelastic haemostatic assay augmented protocols for major trauma haemorrhage (ITACTIC): a randomized, controlled trial. Intensive Care Med. (2021) 47(1):49–59. 10.1007/s00134-020-06266-1 33048195 PMC7550843

[7] Akay OM Ustuner Z Canturk Z Mutlu FS Gulbas Z. Laboratory investigation of hypercoagulability in cancer patients using rotation thrombelastography. Med Oncol. (2009) 26(3):358–64. 10.1007/s12032-008-9129-0 19021004

[8] National Health Commission of the People’s Republic of China. Guidelines for the diagnosis and treatment of Mycoplasma pneumonia in children (2023 edition). Int J Epidemiol Infect Dis. (2023) 50(2):79–85. 10.3760/cma.j.cn331340-20230217-00023

[9] National Health Commission of the People’s Republic of China, State Administration of Traditional Chinese Medicine. Standards for diagnosis and treatment of community-acquired pneumonia in children (2019 edition). Chin J Clin Infect Dis. (2019) 12(1):6–13. 10.3760/cma.j.issn.1674-2397.2019.01.002

[10] Tong L Huang S Zheng C Zhang Y Chen Z. Refractory *Mycoplasma pneumoniae* pneumonia in children: early recognition and management. J Clin Med. (2022) 11(10):2824. 10.3390/jcm11102824 35628949 PMC9144103

[11] Hartmann J Hermelin D Levy JH. Viscoelastic testing: an illustrated review of technology and clinical applications. Res Pract Thromb Haemost. (2023) 7(1):100031. 10.1016/j.rpth.2022.100031 36760779 PMC9903681

[12] Adelborg K Larsen JB Hvas AM. Disseminated intravascular coagulation: epidemiology, biomarkers, and management. Br J Haematol. (2021) 192(5):803–18. 10.1111/bjh.17172 33555051

[13] Zhang Y Zhou Y Li S Yang D Wu X Chen Z. The clinical characteristics and predictors of refractory Mycoplasma pneumoniae pneumonia in children. PLoS One. (2016) 11(5):e0156465. 10.1371/journal.pone.0156465 27227519 PMC4882022

[14] Levi M van der Poll T. Coagulation and sepsis. Thromb Res. (2017) 149:38–44. 10.1016/j.thromres.2016.11.007 27886531

[15] Semeraro N Ammollo CT Semeraro F Colucci M. Sepsis-associated disseminated intravascular coagulation and thromboembolic disease. Mediterr J Hematol Infect Dis. (2010) 2(3):e2010024. 10.4084/mjhid.2010.024 21415977 PMC3033145

[16] Iba T Helms J Connors JM Levy JH. The pathophysiology, diagnosis, and management of sepsis-associated disseminated intravascular coagulation. J Intensive Care. (2023) 11(1):24. 10.1186/s40560-023-00672-5 37221630 PMC10202753

[17] Milbrandt EB Reade MC Lee M Shook SL Angus DC Kong L Prevalence and significance of coagulation abnormalities in community-acquired pneumonia. Mol Med. (2009) 15(11-12):438–45. 10.2119/molmed.2009.00091 19753144 PMC2743205

[18] Davis MD Rubin BK. Plastic Bronchitis. In: Goldfarb S, Piccione J, editors. Diagnostic and Interventional Bronchoscopy in Children. Respiratory Medicine. Cham: Humana (2021). p. 289–93. 10.1007/978-3-030-54924-4_23

[19] Krafft C Christy C. *Mycoplasma* pneumonia in children and adolescents. Pediatr Rev. (2020) 41(1):12–9. 10.1542/pir.2018-0016 31894069

